# *Mycoplasma pneumoniae* Community-Acquired Respiratory Distress Syndrome Toxin Uses a Novel KELED Sequence for Retrograde Transport and Subsequent Cytotoxicity

**DOI:** 10.1128/mBio.01663-17

**Published:** 2018-01-23

**Authors:** Kumaraguruparan Ramasamy, Sowmya Balasubramanian, Krishnan Manickam, Lavanya Pandranki, Alexander B. Taylor, P. John Hart, Joel B. Baseman, T. R. Kannan

**Affiliations:** a Department of Microbiology, Immunology and Molecular Genetics, University of Texas Health Science Center at San Antonio, San Antonio, Texas, USA; b X-Ray Crystallography Core Laboratory, Institutional Research Cores and Department of Biochemistry and Structural Biology, University of Texas Health Science Center at San Antonio, San Antonio, Texas, USA; c Department of Veterans Affairs, South Texas Veterans Health Care System, San Antonio, Texas, USA; Harvard Medical School

**Keywords:** CARDS toxin, KDEL, KELED, *Mycoplasma*, retrograde transport, vacuolation

## Abstract

*Mycoplasma pneumoniae* is an atypical bacterium that causes respiratory illnesses in humans, including pharyngitis, tracheobronchitis, and community-acquired pneumonia (CAP). It has also been directly linked to reactive airway disease, asthma, and extrapulmonary pathologies. During its colonization, *M. pneumoniae* expresses a unique ADP-ribosylating and vacuolating cytotoxin designated community-acquired respiratory distress syndrome (CARDS) toxin. CARDS toxin persists and localizes in the airway in CAP patients, asthmatics, and trauma patients with ventilator-associated pneumonia. Although CARDS toxin binds to specific cellular receptors, is internalized, and induces hyperinflammation, histopathology, mucus hyperplasia, and other airway injury, the intracellular trafficking of CARDS toxin remains unclear. Here, we show that CARDS toxin translocates through early and late endosomes and the Golgi complex and concentrates at the perinuclear region to reach the endoplasmic reticulum (ER). Using ER-targeted SNAP-tag, we confirmed the association of CARDS toxin with the ER and determined that CARDS toxin follows the retrograde pathway. In addition, we identified a novel CARDS toxin amino acid fingerprint, KELED, that is required for toxin transport to the ER and subsequent toxin-mediated cytotoxicity.

## IMPORTANCE

*Mycoplasma pneumoniae*, a leading cause of bacterial community-acquired pneumonia (CAP) among children and adults in the United States, synthesizes a 591-amino-acid ADP-ribosylating and vacuolating protein, designated community-acquired respiratory distress syndrome (CARDS) toxin. CARDS toxin alone is sufficient to induce and mimic major inflammatory and histopathological phenotypes associated with *M. pneumoniae* infection in rodents and primates. In order to elicit its ADP-ribosylating and vacuolating activities, CARDS toxin must bind to host cell receptors, be internalized via clathrin-mediated pathways, and subsequently be transported to specific intracellular organelles. Here, we demonstrate how CARDS toxin utilizes its unique KELED sequence to exploit the retrograde pathway machinery to reach the endoplasmic reticulum (ER) and fulfill its cytopathic potential. The knowledge generated from these studies may provide important clues to understand the mode of action of CARDS toxin and develop interventions that reduce or eliminate *M. pneumoniae*-associated airway and extrapulmonary pathologies.

## INTRODUCTION

*Mycoplasma pneumoniae* is an atypical bacterial pathogen with a streamlined genome that is implicated in a range of acute and chronic respiratory illnesses in humans. It is the major cause of bacterium-associated community-acquired pneumonia (CAP) in the United States, especially among children (1–3), and can lead to reactive airway disease, including asthma, and to numerous extrapulmonary manifestations (4–7). Still, the mechanisms by which *M. pneumoniae* mediates host cell injury have been elusive.

In 2006, we identified a novel 591-amino-acid ADP-ribosylating and vacuolating toxin synthesized by *M. pneumoniae* that we designated community-acquired respiratory distress syndrome (CARDS) toxin (8). The N-terminal 200 residues of CARDS toxin share homology with the cholera toxin (CT) and pertussis toxin (PT) S1 subunit, including the signature sequence motif that is characteristic of the family of CT-like ADP-ribosyltransferase (ADPRT) toxins (9, 10). In contrast to the multisubunit nature of CT and PT, CARDS toxin occurs as a single polypeptide chain, like diphtheria toxin (DT). In addition to ADPRT activity, CARDS toxin induces cellular vacuolization, exfoliation of mucosal cells, and ciliostasis (8, 11). Importantly, in murine and primate models, CARDS toxin alone induces proinflammatory cytokines, lymphocyte activation, and airway pathologies and dysfunction that recapitulate the characteristic symptoms of *M. pneumoniae* infection (12). We further showed that CARDS toxin induces interleukin-1β (IL-1β) release upon ADP-ribosylation of NLRP3, a major inflammasome protein (13). Consistent with this observation, we recently reported the role of NLRP3 as a critical mediator of inflammation during *M. pneumoniae* infection (14). Although *M. pneumoniae* synthesizes very limited amounts of CARDS toxin during growth in laboratory medium, toxin production is markedly upregulated during infection of mice (15). This is also reflected in humans, as CARDS toxin is readily detected in respiratory samples from *M. pneumoniae-*infected individuals (5, 16, 17).

Functional domain analysis of CARDS toxin confirmed the association of the ADP-ribosylating activity with its N terminus and the vacuolating activity and receptor-binding functions with its C terminus (10). Also, our recent three-dimensional structural analysis of CARDS toxin revealed the presence of three distinct domains. Domain 1 (D1) houses the ADP-ribosylating activity, and domains 2 (D2) and 3 (D3) are responsible for receptor recognition, binding, internalization, and vacuolization (9). To exert its ADP-ribosylating and vacuolating activities, CARDS toxin binds to and is internalized via at least two protein receptors on the plasma membrane of susceptible host cells, surfactant protein A and annexin A2 (18, 19). In addition, we demonstrated that CARDS toxin selectively binds to phosphatidylcholine and sphingomyelin (9). Upon binding to these receptors, CARDS toxin is endocytosed primarily by the clathrin-mediated pathway (20).

Bacterial toxins exploit multiple routes to reach their intracellular targets. Some toxins, such as DT and botulinum toxin, traffic through the cytosol by translocating across the membrane at the endosomal level in a pH-dependent manner (21–25). Other bacterial toxins, including Shiga toxin (ST), CT, and *Escherichia coli* heat-labile enterotoxin (LT), utilize the retrograde transport pathway to reach the endoplasmic reticulum (ER) (26–28).

To date, little is known about the entry and trafficking of *M. pneumoniae* CARDS toxin. In this study, we utilized organelle-specific antibodies and SNAP-tag methodologies to identify and characterize toxin entry and transport within targeted cells. Our findings reveal interesting and unique structural and biological attributes of CARDS toxin that enable its intracellular trafficking and compartmentalization, leading to ADP-ribosylation-mediated IL-1β release and vacuole genesis.

## RESULTS

### CARDS toxin intracellular distribution is temperature and time dependent.

**FIG 1:**
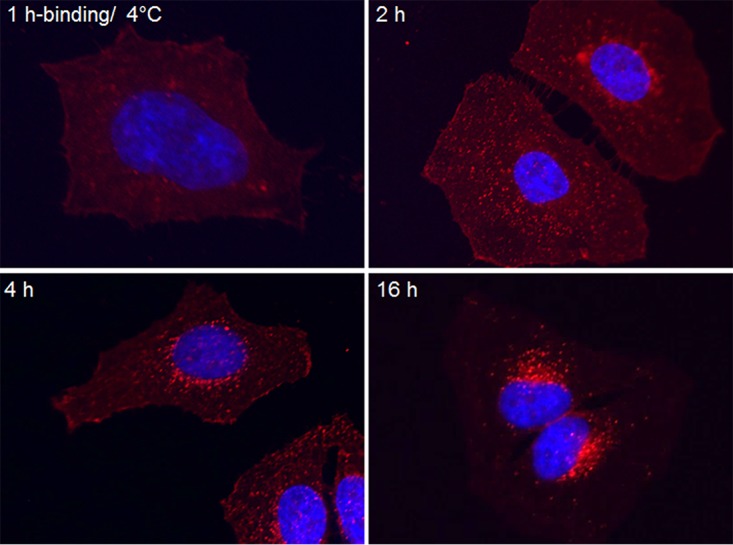
Binding and time-dependent intracellular transport of CARDS toxin in HeLa cells. HeLa cells were treated with 140 pmol of CARDS toxin for 1 h at 4°C. After 1 h of incubation at 4°C, unbound CARDS toxin was removed by washing, cells were shifted to 37°C in fresh medium, and incubation continued up to 16 h. At specific time points, as described in Materials and Methods, cells were fixed, permeabilized, incubated with rabbit anti-CARDS toxin antibody (1:1,000), and stained with Alexa Fluor 555-conjugated secondary antibody (1:1,000) to detect cellular binding and internalization of CARDS toxin (red) by immunofluorescence microscopy. Cell nuclei were stained with DAPI (blue).

HeLa cells were treated with CARDS toxin, and its subsequent distribution was followed by immunofluorescence (IF) microscopy using toxin-specific polyclonal antibodies. As we reported previously (20), upon incubation at 4°C, CARDS toxin remained localized to the cell surface. Two hours after shifting to 37°C, however, the toxin was observed as bright fluorescent puncta throughout the cytoplasm, indicating its uptake into vesicular structures (Fig. 1). Four hours postincubation, internalized CARDS toxin trafficked toward the nucleus, and at 16 h, most of the toxin concentrated around the perinuclear region (Fig. 1).

### CARDS toxin uses retrograde pathway and reaches endoplasmic reticulum.

**FIG 2:**
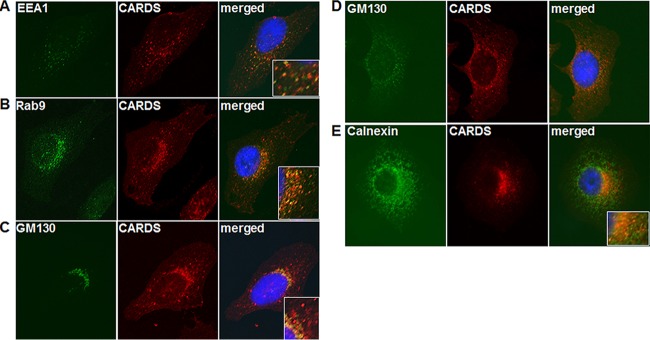
Trafficking of CARDS toxin through early and late endosomes and the Golgi complex to reach the ER. HeLa cells were treated with CARDS toxin at 4°C as described in the legend to Fig. 1, shifted to 37°C, incubated for 15 min to 8 h, and fixed and processed for coimmunofluorescence analysis (co-IFA). Early endosomes were labeled using mouse anti-EEA1 monoclonal antibody (1:100), late endosomes using anti-Rab9 antibody (1:100), Golgi complex using anti-GM130 antibody (1:1,000), and ER using anticalnexin antibody (1:100). The primary antibodies were counterstained using secondary antibody conjugated with Alexa Fluor 488 (green; 1:1,000). CARDS toxin was labeled with rabbit anti-CARDS toxin polyclonal antibody (1:1,000) and stained with Alexa Fluor 555-conjugated secondary antibody (red; 1:1,000). Nuclei were stained with DAPI (blue). Images were collected sequentially from different channels by immunofluorescence microscopy. The merged images show colocalization of CARDS toxin with different organelles. The boxed insets in the merged panels show magnified views of both colocalized and distinct patterns of CARDS toxin and specific organelle staining. (A) EEA1 and CARDS toxin at 1 h. (B) Rab9 and CARDS toxin at 2 h. (C) GM130 and CARDS toxin at 4 h. (D) Cells pretreated with BFA (10 μg/ml), incubated with CARDS toxin for 4 h, and immunostained for GM130 and CARDS toxin. (E) Calnexin and CARDS toxin at 8 h.

HeLa cells were treated with CARDS toxin for designated time periods (15 to 120 min) to evaluate its early endosomal association using antibodies reactive against early endosome antigen 1 (EEA1). Immunofluorescence analysis (IFA) of EEA1 (Fig. 2A, green) and CARDS toxin (Fig. 2A, red) revealed their distribution as individual puncta throughout the cytoplasm. Merged images of EEA1 and CARDS toxin demonstrated their colocalization within 30 min of internalization (Fig. 2A, yellow puncta in merged panel and inset). The kinetics of colocalization was determined by quantifying the number of CARDS toxin-positive pixels associated with EEA1-defined early endosomes. Within 15 min of internalization, the majority of CARDS toxin-positive puncta overlapped with EEA1 (52% ± 8%). Subsequently, by 1 h, the levels of CARDS toxin in the early endosomes waned to around 36% ± 12%, possibly indicating trafficking of CARDS toxin to other vesicular structures. To determine the relationship between CARDS toxin and late endosomal vesicles, we used antibodies against the small GTPase Rab9, which contributes to the generation and maintenance of late endocytic compartments (29). As shown by the results in Fig. 2B, CARDS toxin colocalized with Rab9-enriched puncta within 30 min (47% ± 9%) and 60 min (50% ± 7%), reinforcing the close association of the toxin with late endosomal vesicles (note the abundance of yellow puncta in the merged panel and inset in Fig. 2B). The colocalization of CARDS toxin with the late endosome marker Rab9 increased upon longer incubation (60% ± 7%; 120 min), indicating that CARDS toxin moves sequentially from early endosomes to late endosomes. Under these conditions, very little CARDS toxin colocalized with the Golgi complex (18% ± 4%). However, by 4 h, the majority of internalized toxin was detected within the Golgi complex (80% ± 7%), as it overlapped with the *cis*-Golgi marker GM130 (Fig. 2C, merged panel and inset). To further confirm the association of CARDS toxin with the Golgi complex, toxin distribution was evaluated in the presence of brefeldin A (BFA), a compound that causes disassembly of the Golgi complex and accumulation of secretory proteins in the ER (30). Treatment of cells with BFA (Fig. 2D) led to a more diffuse staining pattern for both CARDS toxin and GM130 protein, confirming that BFA disruption of the Golgi apparatus altered CARDS toxin distribution, consistent with the intimate interplay between CARDS toxin and the Golgi complex. To further examine the relationship between ER and CARDS toxin, we used antibody against calnexin, a transmembrane molecular chaperone involved in the folding and quality control of glycoproteins synthesized in the ER. Eight hours post-toxin uptake, CARDS toxin colocalized with calnexin (50% ± 7%) (Fig. 2E, red, green, and yellow indicate CARDS toxin, calnexin, and colocalization, respectively). This signal overlap was also readily visible at 4 h postentry (30% ± 11%), suggesting that the majority of toxin had reached the ER between 4 and 8 h postintoxication. Similar studies with human airway A549 cells also revealed CARDS toxin transport to the Golgi complex (see Fig. S1 in the supplemental material).

10.1128/mBio.01663-17.1 FIG S1  Intracellular transport of CARDS toxin in A549 cells. A549 cells were treated with CARDS toxin at 4°C as described in the legend to Fig. 1, shifted to 37°C, incubated for 4 h, and fixed and processed for co-IFA. CARDS toxin was labeled with rabbit anti-CARDS toxin polyclonal antibody (1:1,000) and stained with Alexa Fluor 555-conjugated secondary antibody (red; 1:1,000). The Golgi complex was labeled using anti-GM130 antibody (1:1,000) and counterstained using secondary antibody conjugated with Alexa Fluor 488 (green; 1:1,000). Nuclei were stained with DAPI (blue). The merged images showed colocalization of CARDS toxin with the Golgi complex. Download FIG S1, TIF file, 0.2 MB. Copyright © 2018 Ramasamy et al. 2018 Ramasamy et al. This content is distributed under the terms of the Creative Commons Attribution 4.0 International license.

### CARDS toxin possesses a unique ER retrieval sequence.

**FIG 3:**
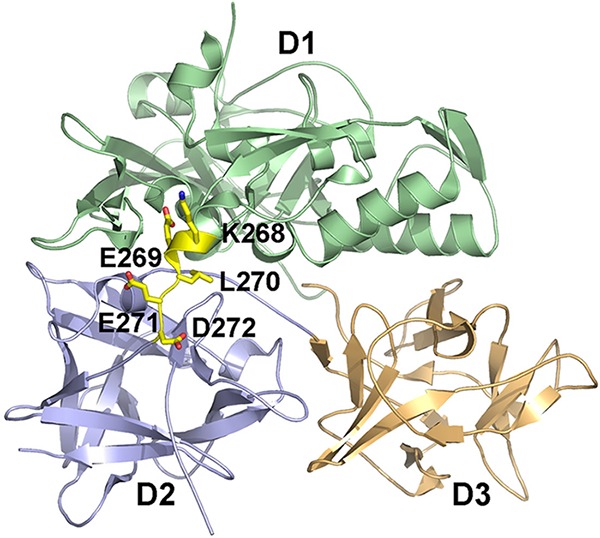
KELED motif in CARDS toxin. The surface-exposed ^268^KELED^272^ motif linking the D1 and D2 domains of CARDS toxin is shown in yellow. The D1, D2, and D3 domains are colored light green, light blue, and light orange, respectively. The figure was prepared using PyMOL (The PyMOL Molecular Graphics System, version 1.8; Schrödinger, LLC).

Analysis of the CARDS toxin amino acid sequence revealed the presence of a KELED sequence at amino acids 268 to 272 (^268^KELED^272^), reminiscent of the KDEL motif involved in targeting intracellular proteins to the ER (31). The crystal structure of CARDS toxin indicated that the KELED sequence was located in the solvent-accessible linker between the D1 ADPRT domain of CARDS toxin and the C-terminal cell surface receptor-binding domains D2 and D3 (Fig. 3).

### CARDS toxin localizes to the ERGIC.

**FIG 4:**
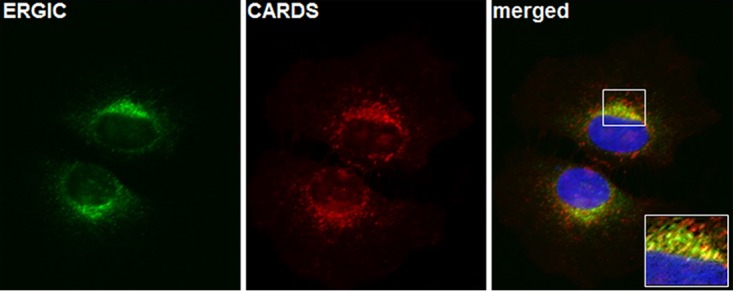
Trafficking of CARDS toxin to the ER through the ERGIC. HeLa cells were treated with CARDS toxin for 8 h at 37°C, fixed, and processed for co-IFA using anti-ERGIC-53 antibody (green) followed by rabbit anti-CARDS polyclonal antibody (red). The merged image shows colocalization of CARDS toxin and ERGIC around the perinuclear region (yellow).

Because proteins involved in the retrieval of escaped ER proteins localize at the integral membrane of the ER-Golgi intermediate compartment (ERGIC) (32), we tested the physical relationship between CARDS toxin and the ERGIC using ERGIC-53-specific antibody; ERGIC-53 is a mannose-specific ERGIC-associated membrane lectin. Immunofluorescence analysis of cells 4 h postintoxication showed similar vesicular fluorescence patterns for ERGIC-53 and CARDS toxin, with resultant colocalization (Fig. 4, green, red, and yellow).

### Mutation of KELED sequence alters CARDS toxin transport to the ER.

**FIG 5:**
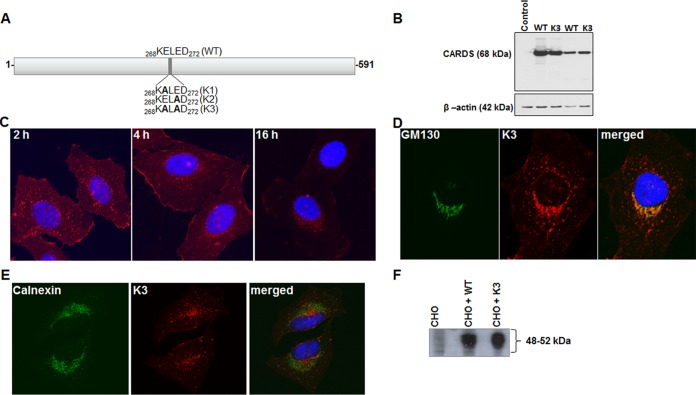
Binding, internalization, trafficking, and ADP-ribosylating activities of K3 CARDS toxin derivative. (A) Schematic representation of location of site-directed mutations at KE^269E→A^LE^271E→A^D (K3). (B) HeLa cells were treated with 280 pmol of WT or mutant K3 CARDS toxin at 4°C for 1 h (binding), followed by washing and incubating at 37°C for 1 h (uptake). Then, cells were harvested, and cell lysates were resolved by SDS-PAGE, transferred to nitrocellulose membrane, and probed with rabbit anti-CARDS toxin antibody. Comparative β-actin intensities were used as loading controls. (C) Binding and internalization of K3 CARDS toxin in HeLa cells. Immunofluorescence analysis was performed as described in the legend to Fig. 1. (D) Co-IFA of K3 CARDS toxin and GM130. HeLa cells were treated with K3 CARDS toxin for 4 h at 37°C, fixed, and stained with anti-GM130 antibody as described in the legend to Fig. 3. (E) Co-IFA of K3 CARDS toxin and calnexin. HeLa cells were treated with K3 CARDS toxin for 8 h at 37°C, fixed, and stained with anti-calnexin antibody as described in the legend to Fig. 4. (F) Comparison of ADP-ribosylating activities of WT and K3 CARDS toxin. WT or K3 CARDS toxin was incubated with CHO cell lysate in the presence of [^32^P]NAD as described in Materials and Methods, and the radiolabeled, ADP-ribosylated, CHO cell target proteins were detected by autoradiogram.

To determine the role of the KELED sequence as a mediator of trafficking to the ER, we generated three CARDS toxin constructs by changing the nucleotides encoding glutamic acid at position 269, 271, or both to nucleotides encoding alanine by site-directed mutagenesis (KE→ALED, KELE→AD, and KE→ALE→AD; mutated amino acids are underlined) (Fig. 5A). The resulting constructs, KALED (K1), KELAD (K2), and KALAD (K3), were analyzed for their effects on CARDS toxin trafficking patterns. No difference was observed in terms of the binding or internalization of wild-type (WT) and K3 CARDS toxin (Fig. 5B). Similar to the WT, K3 CARDS toxin also reached the perinuclear region (Fig. 1 and 5C). However, colocalization studies with organelle-specific antibodies indicated the presence of K3 in the Golgi apparatus (Fig. 5D) but markedly lower levels in the ER (Fig. 5E). The K1 and K2 trafficking patterns were indistinguishable from that of WT CARDS toxin (data not shown). To validate the functional ADP-ribosylating enzymatic property of K3 CARDS toxin, we used CHO cell lysates, and we observed a pattern of ADP-ribosylated target proteins similar to that of WT CARDS toxin (Fig. 5F) (8).

**FIG 6:**
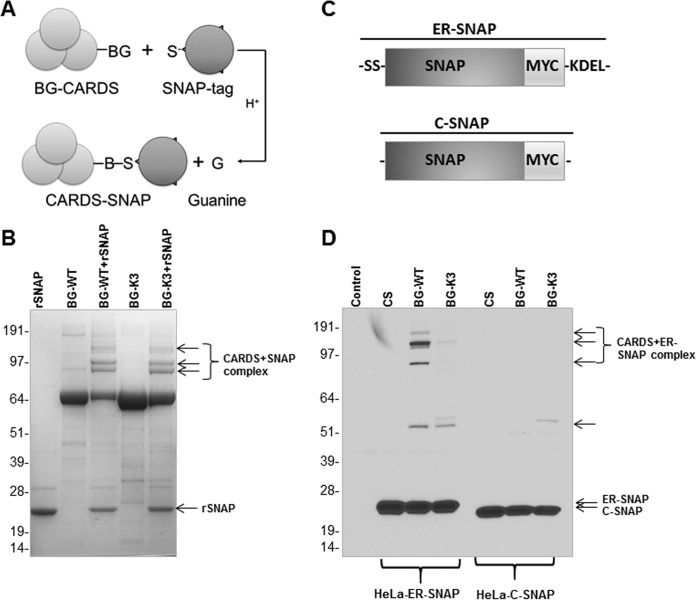
Differences between interactions of WT CARDS toxin and K3 CARDS toxin derivative with the ER and SNAP-tag. (A) Schematic representation of interaction of benzylguanine (BG)-labeled CARDS toxin with SNAP-tag protein; illustration adapted from Geiger et. al. (33). (B) BG-labeled WT CARDS toxin (BG-WT) and K3 CARDS toxin (BG-K3) proteins were individually incubated with purified recombinant SNAP‐tag protein (BG-WT+rSNAP and BG-K3+rSNAP, respectively) for 1 h at 37°C and analyzed by SDS-PAGE followed by Coomassie brilliant blue staining. As controls, SNAP‐tag, BG‐WT CARDS toxin, and BG-K3 CARDS toxin were analyzed. Arrows indicate the complex formed between BG CARDS toxin and SNAP-tag protein. (C) Schematic representation of C-SNAP (SNAP-tag protein coupled with c-Myc at the C terminus) and ER-SNAP (C-SNAP coupled with signal sequence [SS] at the N terminus and KDEL at the C terminus). (D) HeLa cells transfected with c-Myc-tagged ER-SNAP or C-SNAP were incubated with BG-WT or BG-K3 CARDS toxin or toxin carrier solution (CS) for 16 h, lysed, and resolved by SDS-PAGE. Gels were transferred to nitrocellulose membrane and probed with antibody specific to the c-Myc epitope. Arrows indicate the BG-CARDS-SNAP complexes. C-SNAP served as internal quality control. (B, D) Numbers to the left indicate molecular size in kDa.

To further confirm the altered trafficking pattern of K3 CARDS toxin, we used a novel method called ER-SNAP that permits the direct capture of proteins associated with the ER. SNAP-tag is an engineered variant of the human repair protein *O*^6^-alkylguanine-DNA alkyltransferase (hAGT) that covalently reacts with benzylguanine (BG) derivatives (33). CARDS toxin (WT or K3) was coupled to the benzyl moiety through its lysine residues and covalently bonded to the SNAP-tag (Fig. 6A). SDS-PAGE gel mobility shift assays demonstrated that SNAP-tag proteins formed covalent linkage products with the BG-labeled WT CARDS toxin (BG-WT CARDS toxin) or BG-K3 CARDS toxin that were absent when SNAP-tag, BG-WT CARDS toxin, and BG-K3 CARDS toxin were incubated alone (Fig. 6B). Among the distinct bands observed, the faster-migrating bands (≈88 kDa) suggest linkages between CARDS toxin variants (BG-WT or BG-K3 CARDS toxin; 68 kDa) and SNAP-tag (20 kDa), while the more slowly migrating (high-molecular-mass) bands could be interactions between toxin variants and two or more SNAP-tag proteins, which would be dependent on the number of CARDS toxin lysine residues that were labeled with BG.

In order to further define the ER trafficking of CARDS toxin, HeLa cells were transfected with either Myc-tagged ER-SNAP (Fig. 6C, HeLa-ER-SNAP) or Myc-tagged cytosol-SNAP (C-SNAP) (Fig. 6C, HeLa-C-SNAP), which restrict the localization of the SNAP-tag to either the ER or cytosol, respectively. Specifically, ER-SNAP will localize to the ER because it possesses an ER signal sequence in the N terminus and the KDEL retention sequence at the C terminus (Fig. 6C). In contrast, C-SNAP will be unable to traffic to the ER because it lacks the ER signal sequence and retains only c-Myc. Therefore, we hypothesized that if CARDS toxin traffics to the ER, we should detect a BG-WT CARDS toxin–ER-SNAP complex. HeLa cells transfected with ER-SNAP were exposed to BG-WT or BG-K3 CARDS toxin for 16 h, and cell lysates were analyzed by immunoblotting using anti-c-Myc tag antibodies (Fig. 6D). In addition to unreacted ER-SNAP, running at a molecular size of 22 kDa, four reaction products between ER-SNAP and BG-WT CARDS toxin were detected after exposure of cells to CARDS toxin (Fig. 6D, third lane). The reaction products migrated in a range between ≈50 and 191 kDa, indicating that multiple ER-SNAP proteins reacted with BG-WT CARDS toxin and its processed derivatives. Importantly, BG-K3 CARDS toxin exhibited very little association with the ER, despite the high concentration of ER-SNAP (Fig. 6D, fourth lane). As a cytoplasmic control, we exposed BG-labeled WT and K3 CARDS toxins to HeLa cells transfected with C-SNAP. As seen by the results in Fig. 6D, BG-WT and BG-K3 CARDS toxins did not react with C-SNAP, as evidenced by the absence of reactive products. As there are no lysine residues within the ADPRT region of CARDS toxin (until residue 246) for BG labeling, it is possible that this region was not recognized by C-SNAP upon release into the cytosol (Fig. 6D). Together, these data indicate that CARDS toxin reaches the ER using the KELED sequence. As in the c-Myc staining (Fig. 6D; Fig. S2A), the ER-SNAP-transfected cells subjected to BG-labeled CARDS toxin were treated with anti-CARDS toxin antibodies to analyze the CARDS toxin–ER-SNAP complex (Fig. S2B). The detection of high-molecular-mass bands reinforces the idea that ER-SNAP and CARDS toxin will interact. Furthermore, immunoblotting revealed the presence of BG-free CARDS toxin and its processed derivatives (Fig. S2B).

10.1128/mBio.01663-17.2 FIG S2  Analysis of CARDS toxin transport to the ER using ER-SNAP. HeLa cells transiently transfected with ER-SNAP were exposed to BG-CARDS toxin (700 pmol) for 24 h. Cells were harvested at the indicated time points, lysed, and analyzed by SDS-PAGE followed by immunoblot analysis using an antibody against the Myc epitope of ER-SNAP (A) or stripped and reprobed with a polyclonal antibody against CARDS toxin (B). Solid arrows indicate ER-SNAP and BG-CARDS toxin complexes. Dotted arrows indicate BG-free CARDS toxin and its processed form. Download FIG S2, TIF file, 0.1 MB. Copyright © 2018 Ramasamy et al. 2018 Ramasamy et al. This content is distributed under the terms of the Creative Commons Attribution 4.0 International license.

### CARDS toxin trafficking to the ER is essential to execute its cytopathic effects.

**FIG 7:**
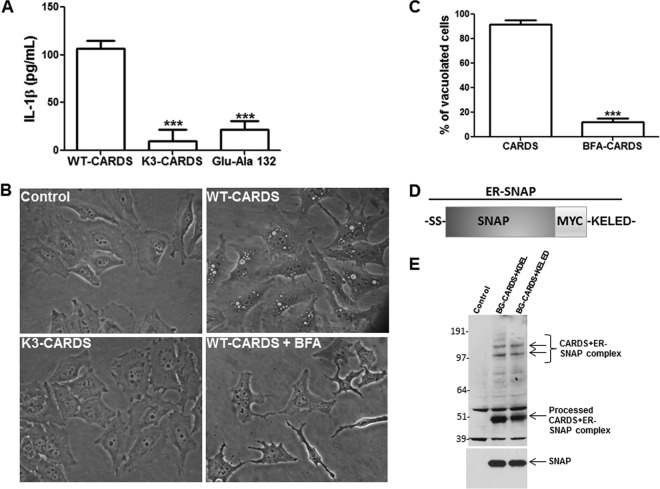
Role of KELED sequence in ER trafficking and subsequent CARDS toxin-induced IL-1β secretion and vacuolation. (A) Human U937 cells were treated with LPS (1 µg/ml) for 4 h and then with 700 pmol of WT CARDS toxin, K3 CARDS toxin, or E132A CARDS toxin for 48 h, and IL-1β levels in medium supernatants were measured by ELISA. Data represent the mean values ± standard deviations from three independent experiments performed in triplicate. *P* values were calculated using analysis of variance (ANOVA) and are indicated as follows: ***, *P* < 0.001. (B) HeLa cells pretreated with or without BFA (10 μg/ml) were incubated with CARDS toxin or K3 CARDS toxin for 12 h at 37°C, and cell images taken. (C) CARDS toxin-mediated vacuolation in BFA-treated HeLa cells. The percentage of inhibition of vacuolation by BFA was evaluated by counting the number of vacuolated cells in BFA-treated cells versus the number in untreated cells. *P* values were calculated using ANOVA and are indicated as follows: ***, *P* < 0.001. (D) Schematic representation of c-Myc-tagged ER-SNAP KELED construct. (E) HeLa cells transfected with c-Myc-tagged ER-SNAP-KDEL, ER-SNAP-KELED, or C-SNAP were incubated with BG-WT CARDS toxin for 16 h, lysed, and resolved by SDS-PAGE. Gels were transferred to nitrocellulose membranes and probed with antibody specific to the c-Myc epitope. Arrows (CARDS) indicate the BG-CARDS-SNAP complexes. C-SNAP served as an internal quality control. Numbers to the left indicate molecular size in kDa.

Since several bacterial toxins that follow the retrograde pathway execute their cytopathic effects only after reaching the ER (34), we investigated the ADP-ribosylating activity of K3 CARDS toxin in human U937 cells by determining the release of IL-1β. As we reported previously (13), ADP-ribosylation of NLRP3 by WT CARDS toxin leads to the release of the proinflammatory cytokine IL-1β (Fig. 7A). In contrast, the ADPRT E132A mutant of CARDS toxin, in which the catalytic glutamic acid (E132) is modified to alanine to abolish ADPRT activity (8, 10), is unable to ADP-ribosylate NLRP3 and release IL-1β (Fig. 7A). Interestingly, despite its *in vitro* ADPRT activity observed in cell lysates (Fig. 5F), K3 CARDS toxin did not induce IL-1β cytokine release from U937 cells (Fig. 7A). Similarly, we analyzed the vacuolating activity of K3 CARDS toxin on HeLa cells. As a control, we used BFA treatment, which is known to disrupt the Golgi complex-ER network and protect cells against bacterial toxins (like PE, ST, LT, and *E. coli* Shiga-like toxin 1) that traffic through the retrograde pathway (35). We found that incubation of K3 CARDS toxin with HeLa cells did not elicit vacuolating activity (Fig. 7B, lower left); even prolonged incubation (48 to 72 h) failed to induce vacuolation. The addition of BFA to HeLa cells prior to CARDS toxin treatment resulted in a 90% decrease in vacuolation compared to that in cells treated with CARDS toxin alone (*P* < 0.001 for the results at 12 h) (Fig. 7B and C). These data provide additional evidence that CARDS toxin trafficking to the ER is required to induce vacuole formation and IL-1β release. To further confirm that BFA treatment affects the time-dependent ER trafficking of CARDS toxin, we treated ER-SNAP-transfected cells with BFA and BG-CARDS toxin and analyzed ER-SNAP and BG-CARDS toxin interactions at 12- to 24-h time intervals. The presence of BFA markedly reduced CARDS toxin trafficking to the ER in a time-dependent manner (Fig. S3).

10.1128/mBio.01663-17.3 FIG S3  Brefeldin A (BFA) blocks CARDS toxin’s association with the Golgi complex. HeLa cells were transiently transfected with ER-SNAP. After 24 h, the cells were treated with BFA (10 µg/ml) for 1 h at 37°C. After 1 h of pretreatment with BFA, the cells were exposed to BG-CARDS toxin (700 pmol) for 12 h and 24 h. Cells were harvested at indicated time points and analyzed by SDS-PAGE followed by immunoblot analysis using an antibody against the Myc epitope of ER-SNAP. Download FIG S3, TIF file, 0.2 MB. Copyright © 2018 Ramasamy et al. 2018 Ramasamy et al. This content is distributed under the terms of the Creative Commons Attribution 4.0 International license.

### KELED sequence mediates transport of proteins to the ER.

To further evaluate the role of the CARDS toxin KELED sequence in promoting protein trafficking to the ER, we generated an ER-SNAP plasmid encoding the KELED sequence (ER-SNAP-KELED) (Fig. 7D), like the ER-SNAP-KDEL plasmid (Fig. 6C), and transfected HeLa cells with it. Immunofluorescence analysis clearly showed localization of the KELED-containing constructs in a fine reticular network at the nuclear envelope (Fig. S4), suggesting that these fusion proteins localized mainly to the ER. To further validate their ER trafficking properties, we compared the interactions of BG-CARDS in ER-SNAP-KELED- or ER-SNAP-KDEL-transfected HeLa cells as described above. We detected high-molecular-mass proteins in the anti-c-Myc tag antibody immunoblots of both ER-SNAP-KELED and ER-SNAP-KDEL but not in the control (Fig. 7E), indicating that CARDS toxin exploits host receptors to reach the ER using its KELED sequence.

10.1128/mBio.01663-17.4 FIG S4  Localization of SNAP in a fine reticular network and at the nuclear envelope. Localization of SNAP-tag protein in ER-SNAP-KDEL- and ER-SNAP-KELED-transfected HeLa cells using SNAP-Cell TMR-Star, a fluorescent substrate that labels SNAP-tag fusion proteins. Cells were fixed, and images were taken using a fluorescence microscope. Download FIG S4, TIF file, 0.1 MB. Copyright © 2018 Ramasamy et al. 2018 Ramasamy et al. This content is distributed under the terms of the Creative Commons Attribution 4.0 International license.

## DISCUSSION

Considerable progress has been made in defining the routes by which microbial toxins are delivered to the ER, and here, we describe how the *M. pneumoniae* CARDS toxin reaches the ER via its unique KELED motif. First, we established that CARDS toxin is transported from early endosomes to late endosomes and then by retrograde transport (Golgi complex and ERGIC) to the ER. Second, we utilized ER-SNAP-tag methodology to define the temporal transport of CARDS toxin. Third, we demonstrated that efficient ER trafficking of CARDS toxin is dependent on the distinct KELED motif located between the D1 and D2 domains of CARDS toxin at amino acids 268 to 272. Finally, our studies show that CARDS toxin trafficking to the Golgi complex-ER network is critical for both ADP-ribosylation-dependent NLRP3-mediated secretion of IL-1β and induction of vacuole formation.

As we reported previously (20), CARDS toxin uses receptor-mediated, clathrin-dependent endocytosis to enter target cells, similarly to Shiga and anthrax toxins (36, 37), and subsequently localizes to early endosomes (Fig. 2A). CARDS toxin was detected in substantial amounts in Rab9-positive vesicles, indicating that it transits through late endosomal pathways to reach the Golgi complex (Fig. 2B to E) and escapes lysosomal degradation. This late endosome trafficking of CARDS toxin appears distinct from the transport mechanisms of most other toxins that follow the retrograde pathway, including ST and ricin, which circumvent late endosomes and are sorted directly from early endosomes to the Golgi apparatus (38–40). Interestingly, CARDS toxin and *Pseudomonas* exotoxin A (PE) share similar trafficking patterns (41). It is possible that exposure to low pH in the late endosomes triggers conformational changes within the CARDS toxin protein structure that facilitate KELED-dependent binding to ER recycling receptor(s). Interestingly, KDEL receptors are hijacked by some protein toxins to enable their transport in a retrograde manner to the ER (35), and these toxins have evolved KDEL-like motifs to play a similar role (42). However, unlike the KDEL sequence location at the C terminus of other bacterial toxins (35, 43), the KELED motif in CARDS toxin is uniquely positioned as part of the solvent-accessible linker region between D1 and D2 (Fig. 3).

The colocalization of CARDS toxin with the ERGIC (Fig. 4) was apparent by IFA, further implicating this mechanism in retrograde transport of CARDS toxin (32). In addition, site-directed mutagenesis of KELED to KALAD (Fig. 5, K3), but not to KALED or KELAD, prevented efficient trafficking of CARDS toxin from the Golgi complex to the ER (Fig. 5E), resulting in the accumulation of substantial amounts of K3 CARDS toxin at the Golgi complex, in contrast to the localization of WT CARDS toxin (Fig. 5D). It is likely that the K3 CARDS toxin derivative lacks signal compatibility for transport from the Golgi complex to the ER. Alternatively, the amino acid changes in K3 CARDS toxin could have altered its conformation, preventing recognition by the ER recycling receptors. Importantly, the successful *in vitro* ADP-ribosylation of host target proteins by K3 CARDS toxin, like that of WT CARDS toxin, indicates that the changes in KELED-associated amino acids do not affect its ADPRT activity in cell lysates (Fig. 5F). Furthermore, in contrast to those of the KALAD motif, the ER localization and vacuolation of KALED or KELAD constructs were similar to those of the WT, indicating that at least one negatively charged amino acid (i.e., glutamic acid) next to leucine is necessary for efficient transfer of CARDS toxin to the ER.

Using ER-SNAP-trap methodology, we were able to delineate how endocytosed CARDS toxin trafficked to the ER (Fig. 6). By expressing ER-SNAP or C-SNAP in HeLa cells (Fig. 6D, intense low-molecular-mass protein bands), we were able to detect the interaction of CARDS toxin with ER-trapped SNAP using anti-c-Myc antibody immunoblot analyses (Fig. 6D, high-molecular-mass bands), strongly indicating that CARDS toxin reached the ER. However, the drastic reduction of K3 CARDS toxin’s interaction with ER-SNAP, as observed by the diminished intensity of high-molecular-mass bands (Fig. 6D), demonstrated that KELED is important for toxin trafficking to the ER. These data affirm the observation that the very limited colocalization of K3 CARDS toxin with calnexin is due to the absence of the KELED sequence. Although marked reduction in K3 CARDS toxin transport to the ER was observed, it was not completely blocked, as seen by the faint high-molecular-mass anti-c-Myc antibody-reactive bands (Fig. 6D), suggesting that this transport mechanism is not the only pathway used by toxins to reach the ER. For example, mutations in the KDEL sequence of the A fragment of CT cause a delay in the action of the toxin but do not ablate its function, suggesting that retrograde transport can occur independently of the KDEL receptor (43). Importantly, unlike WT CARDS toxin, K3 CARDS toxin did not elicit IL-1β secretion (Fig. 7A), implying that the blockage of CARDS toxin transport to the ER prevents the activation/cytosolic release of the ADPRT region and subsequent ADP-ribosylation of NLRP3 and IL-1β release. Furthermore, the K3 CARDS toxin mutant, even at high concentrations and longer incubations, did not elicit vacuole formation, indicating that the trafficking of CARDS toxin to the ER plays a key role in overall CARDS toxin-mediated cytotoxicity (Fig. 7B and C).

Proteins containing ER retention motifs, such as KDEL, are thought to be retrieved back to the ER from the intermediate compartment and the *cis*-Golgi (44). The similarity of the interactions of BG-labeled CARDS toxin (BG-CARDS toxin) with SNAP-KELED and SNAP-KDEL (Fig. 7D and E, high-molecular-mass c-Myc bands) suggests that KELED by itself can act as an ER retention signal. To our knowledge, there are no other reports of bacterial or mammalian proteins that use the KELED sequence for intracellular transport and sublocalization.

Based on these findings, we propose that CARDS toxin evolved to possess a unique sequence that enables its transport from the Golgi complex to the ER and intracellular targets. The unusual KELED sequence and its structural location at the linker of D1 and D2 (Fig. 3) differentiate CARDS toxin from other retrograde bacterial toxins and reinforce the unusualness of the genome-streamlining adaptations that *M. pneumoniae* has achieved among bacterial pathogens.

In summary, this study details the relationship between the retrograde transport and subcompartmentalization of CARDS toxin via the novel KELED motif and its subsequent mode of action. Such information could lead to innovative therapeutic interventions to neutralize the action of CARDS toxin, thus resulting in the interruption or prevention of *M. pneumoniae*-mediated acute and chronic airway diseases and extrapulmonary pathologies. Also, it is well established that bacterial toxins offer versatile tools for probing the complex functions and movement of proteins via early endosomes to direct retrograde transport and ER-perinuclear localization. Thus, CARDS toxin provides an additional tool to investigate such intracellular events by manipulating precise pathways and dictating physiological outcomes.

## MATERIALS AND METHODS

### Bacterial strains, plasmids, and DNA manipulations.

The following vectors and bacterial cells were used to generate wild-type and mutant CARDS toxin proteins: pCR2.1 (TA cloning vector; Invitrogen) and *E. coli* INVαF' (F' *endA1 rec1 hsdR17 supE44 gyrA96 lacZ*ΔM15 [*lacZYA-argF*]) for gene manipulations and pET-19b (N-terminal His_10_ tag, expression vector; Novagen/EMD Biosciences, San Diego, CA) and lipid A-deficient *E. coli* BL21(DE3) (*lpxM* F^−^ *ompT hsdSB* [r_B_^−^ m_B_^−^] *gal dcm*) for protein expression (45).

### Cloning, expression, and purification of various CARDS constructs.

Site-directed mutagenesis was carried out by overlap extension PCR (46) using the primers listed in Table 1. UGA-corrected pET19b encoding CARDS toxin served as the template for amplification of mutant CARDS toxin derivatives. All PCR fragments were cloned into pCR2.1 and subcloned into the pET-19b vector under the T7 promoter using NdeI and BamHI cloning sites. Representative constructs were subjected to nucleotide sequencing (Department of Microbiology, Immunology and Molecular Genetics Nucleic Acids Facility, UT Health San Antonio [UTHSA]), and constructs were transformed into *E. coli* BL21(DE3) for expression studies. WT and mutated CARDS toxin proteins were expressed and purified under native conditions on a large scale as reported previously (8, 10), and the homogeneity of purified proteins was analyzed by resolving them in 4 to 12% NuPAGE gradient gels (Invitrogen) and visualizing them using Coomassie brilliant blue.

**TABLE 1:** Primers used in the study

Plasmid, primer^a^	Sequence (5′→3′)^b^
pET19b-CARDS	
CARDS F	CATATGCCAAATCCTGTTAGATTTGTTTACCGTGTT
CARDS R	GCTGCCGCCGCCGCCGCTGCCAAAGCGATCAAAACCATCTTT
K**A**LED F	GTAAAG**GCA**CTGGAAGATACACCAGTATAC
K**A**LED R	GTATACTGGTGTATCTTCCAG**TGC**CTTTAC
KEL**A**D F	GTAAAGGAACTG**GCA**GATACACCAGTATAC
KEL**A**D R	GTATACTGGTGTATC**TGC**CAGTTCCTTTAC
K**A**L**A**D F	GTAAAG**GCA**CTG**GCA**GATACACCAGTATAC
K**A**L**A**D R	GTATACTGGTGTATC**TGC**CAG**TGC**CTTTAC

pCMV-ER-SNAP	
KELED F	GGACGTCTGCAGCTGATGCCGTGGAGGTCC
KELED R	GCTTCTAGACTAATCTTCCAGTTCCTTCTCGCTTGC

a The forward (F) and reverse (R) primers are identified by the appropriate letter at the end of the primer designation, and amino acids mutated to alanine are in boldface.

b In the primer sequences, the spacer sequences are shown in italics, introduced restriction endonuclease sites are shown by underlining, and the mutations in the nucleotides are shown in boldface.

### Mammalian cell cultures, plasmids, chemical reagents, and antibodies.

Human cervical adenocarcinoma HeLa cells (CCL-2), human alveolar adenocarcinoma A549 cells (CCL-185), human pleural effusion U937 monocyte cells, and Chinese hamster ovarian (CHO; CCL-61) cells were obtained from the American Type Culture Collection (ATCC) and cultured in minimal essential medium (MEM; Invitrogen), F-12K medium (ATCC), and RPMI medium (Invitrogen) with 10% heat-inactivated fetal bovine serum (FBS) (Atlas BioLogicals), penicillin (100 units/ml), and streptomycin (100 mg/ml). U937 cells were induced to differentiate by exposing them (3 × 10^5^ cells/ml) to 50 nM of phorbol 12-myristate 13-acetate (PMA) (Sigma) for 24 h. After 24 h, PMA-containing medium was replaced with fresh complete RPMI medium, and cells were maintained for 48 h before treatments. All cell monolayers were grown under conditions of 5% CO_2_, 95% humidity at 37°C. ER-SNAP-KDEL and C-SNAP were the kind gift of Roger Geiger, Institute for Research in Biomedicine, Bellinzona, Switzerland (33). ER-SNAP-KELED was constructed by replacing the KDEL sequence of ER-SNAP-KDEL with the CARDS toxin KELED sequence. Using ER-SNAP-KDEL as a template and the primers given in Table 1, the ER-SNAP-KELED sequence was amplified and subcloned between the PstI and XbaI restriction sites of the pCMV-ER-SNAP plasmid. Constructs were subjected to nucleotide sequencing to confirm the substituted-nucleotide changes and transfected into HeLa cells for ER-SNAP studies. Brefeldin A (BFA), thymidine, dithiothreitol (DTT), magnesium chloride (MgCl_2_), and trichloroacetic acid (TCA) were purchased from Sigma. BG-GLA-NHS was purchased from New England Biolabs, and Lipofectamine 2000 from Invitrogen. Anti-EEA1 (281.7), anti-ERGIC-53 (C-6), and anti-c-Myc (clone 9E10) antibodies were purchased from Santa Cruz. Antibodies reactive against GM130 and calnexin (AF18) were obtained from Abcam, Inc., and antibody against Rab9 from Novus Biologicals. Affinity-purified Alexa Fluor 488 goat anti-mouse and Alexa Fluor 555 goat anti-rabbit antibodies were purchased from Invitrogen. Rabbit anti-CARDS toxin polyclonal antibody was used as described previously (15, 20).

### ADP-ribosylation assays.

ADP-ribosyltransferase activity assays of WT and K3 constructs were performed as previously described (8, 10). Briefly, CHO cell lysates were incubated with WT and K3 CARDS toxins in a 50-μl reaction mixture (10 mM thymidine, 10 mM DTT, 2.5 mM MgCl_2_, 50 mM Tris [pH 7.4], and 0.2 μM [^32^P]NAD [800 Ci mmol^−1^; PerkinElmer]) at room temperature for 30 min. Proteins were TCA precipitated and centrifuged at 16,000 × *g* for 10 min. Cell pellets were suspended in NuPAGE sample buffer (Invitrogen), separated on NuPAGE 4 to 12% bis-Tris gradient gels (Invitrogen), and transferred onto 0.2-μm nitrocellulose membranes (Protran BA83; Schleicher & Schüll). Individual membranes were exposed to autoradiographic film (Kodak) and developed.

### Vacuolation studies.

HeLa cells were grown to 50 to 60% confluence in 6-well plates, and WT and K3 CARDS toxins (140 to 700 pmol or 10 to 50 µg/ml) were added as described previously (11). Cells were observed for vacuolation for up to 72 h. HeLa cells with carrier buffer solution served as negative controls. For BFA experiments, cells were pretreated with or without BFA (10 µg/ml), and CARDS toxin was added. Cell vacuolation was visualized using inverted light microscopy (Olympus CK40).

### IL-1β cytokine assay.

IL-1β assay was performed essentially as described previously (13). In brief, differentiated U937 cells were treated with lipopolysaccharide LPS-EB (LPS from *E. coli* O111:B4) (1 µg/ml; InvivoGen) for 4 h, and subsequently, cells were incubated with 700 pmol of WT CARDS toxin, K3 CARDS toxin, or E132A CARDS toxin for 48 h. IL-1β levels in medium supernatants were measured using a human-specific enzyme-linked immunosorbent assay (ELISA) kit (eBioscience).

### **Immunofluorescence assays for toxin binding, internalization**, **and colocalization studies.**

HeLa cells (2 × 10^4^ cells/well) were grown in appropriate culture medium on cover glasses at 37°C with 5% CO_2_. For toxin binding and internalization studies, cells were washed and incubated with CARDS toxin (140 pmol) in serum-free culture medium at 4°C for 1 h. Unbound toxin was removed by washing cells three times in serum-free medium, and intoxicated cells were incubated in complete medium for various times (15 min to 16 h) at 37°C. Cells were washed three times with phosphate-buffered saline (PBS) (pH 7.4), fixed with 2% paraformaldehyde for 10 min, permeabilized with 0.2% Triton X-100 for 10 min, and blocked with 1% normal goat serum (NGS; Gibco) in PBS. Then, cells were incubated with rabbit anti-CARDS toxin polyclonal antibody (1:1,000 dilution) in 0.2% NGS in PBS for 1 h, washed three times with 0.2% NGS in PBS, and further incubated with secondary goat anti-rabbit polyclonal antibody (1:100 dilution) labeled with Alexa Fluor 555 (Invitrogen) for 1 h. Cellular F-actin was stained with Alexa Fluor 488-conjugated phalloidin (Invitrogen). Finally, cells were washed with PBS and mounted on glass slides using Vectashield Hard Set mounting medium containing DAPI (4,6-diamidino-2-phenylindole dihydrochloride) stain (Vector Lab). For colocalization studies, cells were incubated with antibodies to EEA1 (1:100), Rab9 (1:100), GM130 (1:1,000), ERGIC (1:100), and calnexin (1:100) diluted in PBS with 0.2% NGS for 1 h. Cells were washed with PBS containing 0.2% NGS and incubated with secondary antibody (Alexa Fluor 488 goat anti-mouse antibody, diluted 1:500) in PBS with 0.2% NGS for 1 h at room temperature (RT). Cells were washed again with PBS and stained with rabbit anti-CARDS toxin polyclonal antibody as indicated above. Test samples were observed with a multichannel acquisition system operated by AxioVision version 4.7.2 using a Carl Zeiss Z.1 Cell Observer microscope, and Z sections were processed using AxioVision deconvolution software and Adobe Photoshop for rotating and cropping images.

### Quantification of colocalization.

Colocalization analyses of CARDS toxins with different cellular markers (EEA1, Rab9, GM130, calnexin, and ERGIC) were performed on images obtained with a Carl Zeiss Z.1 Cell Observer immunofluorescence microscope. The cells were outlined using the outline tool, and the Pearson’s coefficient was determined using the colocalization plugin in AxioVision 4.7.2 software.

### Labeling of WT and K3 CARDS toxins with BG.

BG-GLA-NHS (New England Biolabs) was dissolved in dimethyl sulfoxide and added to WT or K3 CARDS toxin to a final concentration of 1 mM (labeling ratio, 1:20). The reaction mixtures were mixed well for 1 h at RT, and unbound BG-GLA-NHS was quenched with 100 mM Tris HCl (pH 8.0) for 15 min and washed four times with 1× PBS, using a centrifugal filter device (Millipore) with a cutoff of 3,000 nominal molecular weight limit.

### SNAP experiments.

HeLa cells grown on 6-well plates to 50 to 60% confluence were transfected with 5 µg of ER-SNAP (ER-SNAP-KDEL or ER-SNAP-KELED) or C-SNAP plasmids using Lipofectamine 2000. After 24 h posttransfection, cells were intoxicated with 700 pmol of freshly prepared BG-CARDS or BG-K3 CARDS toxin protein. Transfected cells with carrier buffer served as controls. After 16 h, cells were harvested, separated in SDS-PAGE, transferred to nitrocellulose membranes, and probed with anti-c-Myc antibody (1:1,000) or rabbit anti-CARDS toxin polyclonal antibody (1:5,000) (15). The latter recognizes multiple CARDS toxin epitopes distributed throughout the D1, D2, and D3 domains.

### BFA treatment.

ER-SNAP plasmid-transfected or untransfected HeLa cells were pretreated with or without BFA (10 µg/ml) in complete medium for 1 h at 37°C. After BFA treatment, cells were washed in cold serum-free medium, CARDS toxin was added to the cells, and the cultures transferred to 4°C for 1 h. Unbound CARDS toxin was removed by washing cells with cold serum-free medium, and prewarmed complete medium with BFA was added. At specific time intervals (4 h, 8 h, 12 h, 24 h, and 48 h), one group of cells was harvested and analyzed for ER-SNAP interactions of CARDS toxin by Western blotting as described above. Other groups of cells were analyzed for CARDS toxin localization by immunofluorescence staining as described above.
