# Supplementary figures and images for: Mycoplasma pneumoniae Community-Acquired Respiratory Distress Syndrome Toxin Uses a Novel KELED Sequence for Retrograde Transport and Subsequent Cytotoxicity
Source: mBio. 2018 Jan 23;9(1):e01663-17. doi: 10.1128/mBio.01663-17 (PMC5784248; doi:10.1128/mBio.01663-17)

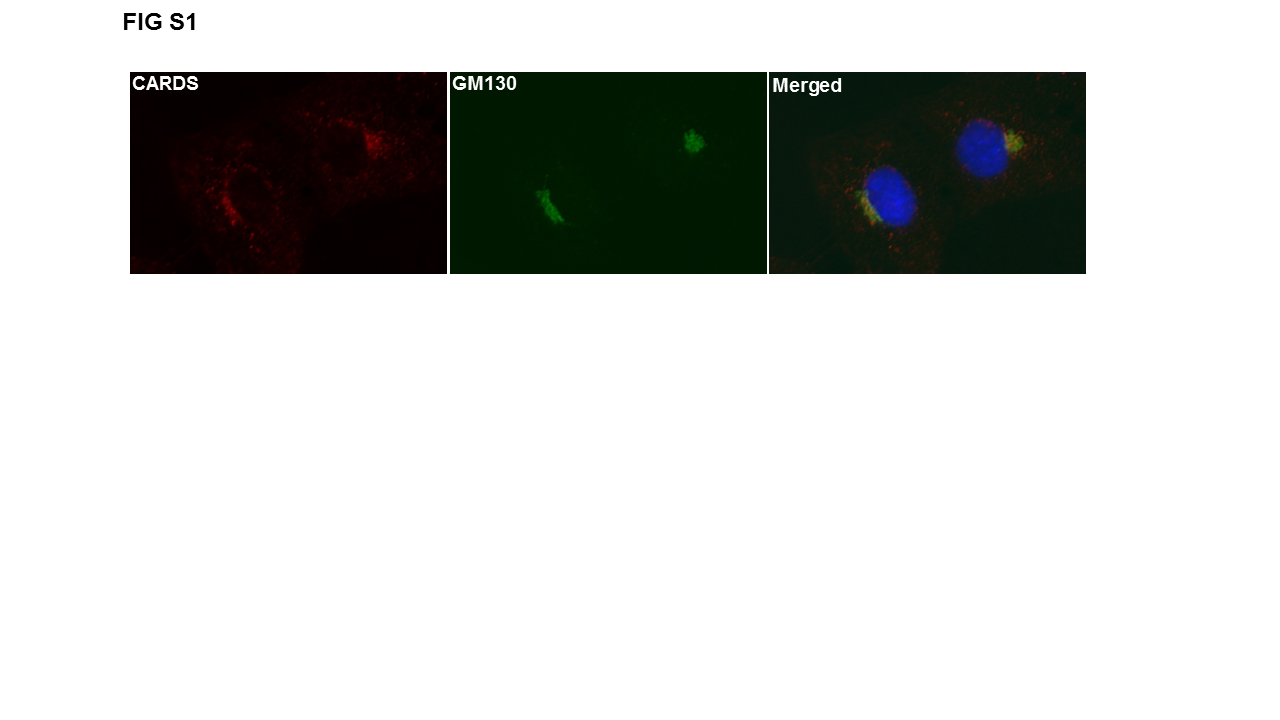

Supplement: FIG S1 [file mbo001183689sf1.tif]

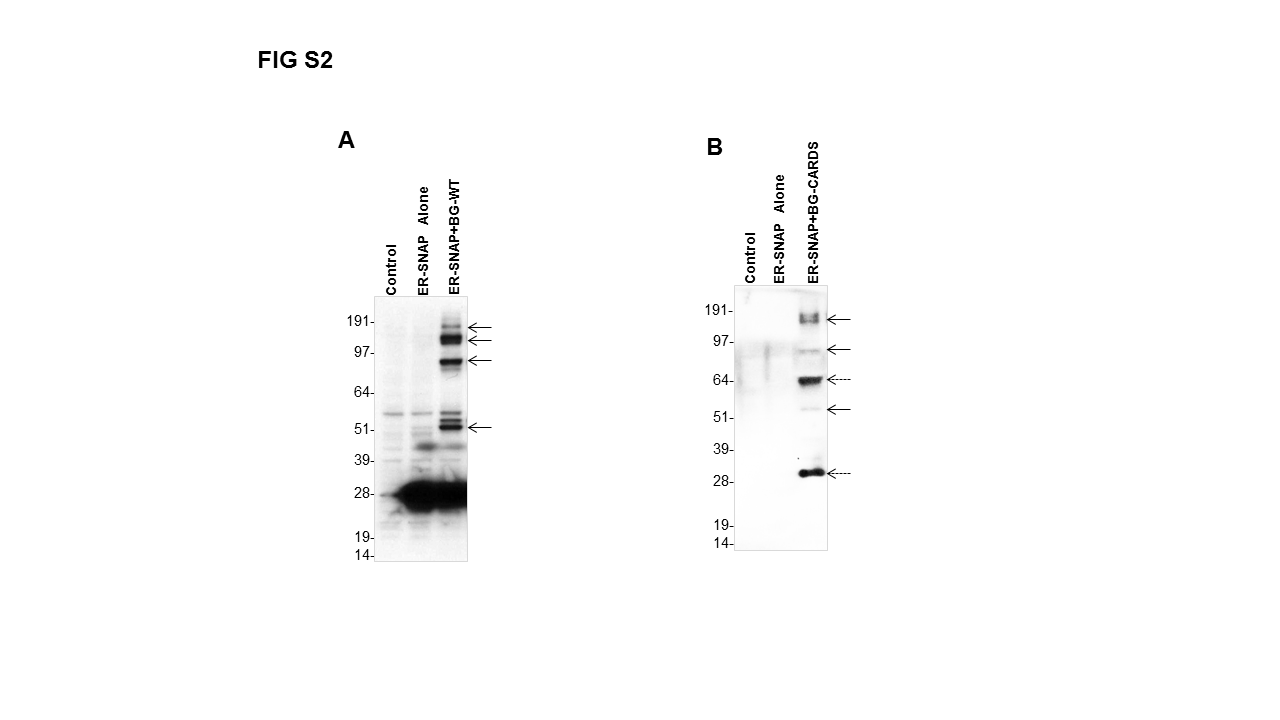

Supplement: FIG S2 [file mbo001183689sf2.tif]

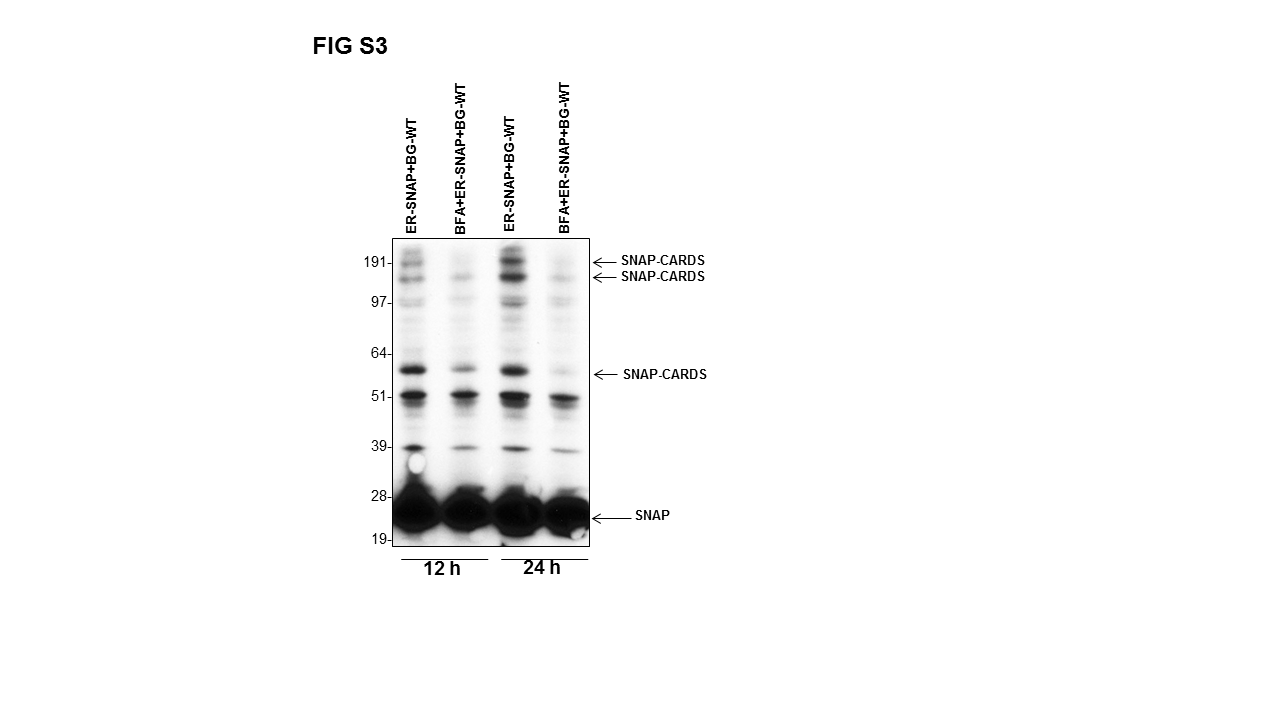

Supplement: FIG S3 [file mbo001183689sf3.tif]

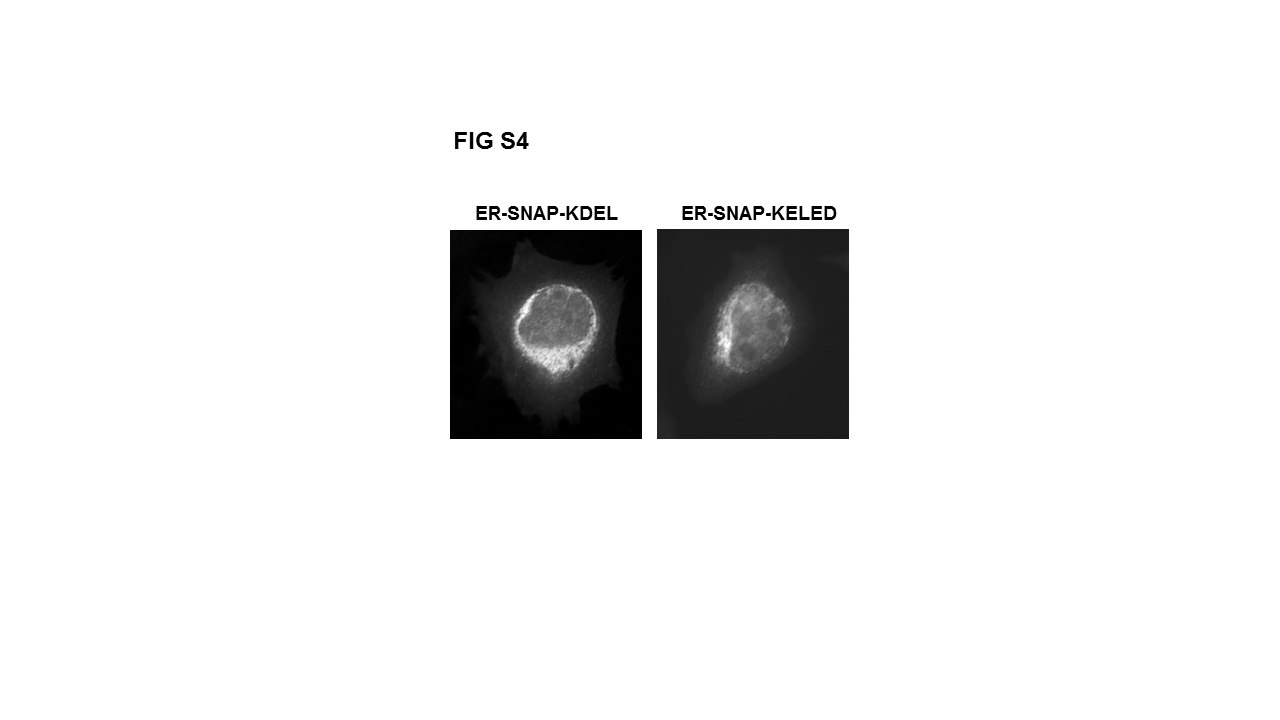

Supplement: FIG S4 [file mbo001183689sf4.tif]
